# Inflammation and Elevation of Interleukin-12p40 in Patients with Schizophrenia

**DOI:** 10.3389/fnmol.2016.00016

**Published:** 2016-03-22

**Authors:** Nora Bedrossian, Mariam Haidar, Jawad Fares, Firas H. Kobeissy, Youssef Fares

**Affiliations:** ^1^Neuroscience Research Center, Faculty of Medical Sciences, Lebanese UniversityBeirut, Lebanon; ^2^Department of Medical Microbiology, Faculty of Medical Sciences, Lebanese UniversityBeirut, Lebanon; ^3^Faculty of Science, Lebanese UniversityBeirut, Lebanon; ^4^Faculty of Medicine, American University of BeirutBeirut, Lebanon; ^5^Department of Psychiatry, College of Medicine and McKnight Brain Institute, University of FloridaGainesville, FL, USA; ^6^Department of Biochemistry and Molecular Genetics, American University of Beirut Medical CenterBeirut, Lebanon; ^7^Department of Neurosurgery, Faculty of Medical Sciences, Lebanese UniversityBeirut, Lebanon

**Keywords:** inflammation, biomarker, schizophrenia, IL-12p40, cytokine, interleukin, ELISA, Lebanon

## Abstract

Schizophrenia is a serious mental illness with chronic symptoms and significant impairment in psychosocial functioning, which suggests that it likely has neurodegenerative characteristics. Inflammatory markers such as pro-inflammatory cytokines are well-known etiological contributors for psychiatric disorders, including schizophrenia. Although, the role of inflammation in schizophrenia is becoming evident, the number of studies in this area is relatively scarce, especially in Lebanon, and increased procedural thoroughness is needed. Cytokines play a key role in the activation of the immune system and strongly influence neurotransmission. Previous investigation of plasma levels showed dysregulation of interleukin (IL)-12. However, genotypical variations of this interleukin have not been investigated for patients with schizophrenia yet. Thus, in this paper, we aimed to compute and assess IL-12p40 levels in the sera of individuals with schizophrenia from different provinces in Lebanon and compare it to controls. Healthy subjects comprised 60 individuals with a male/female (M/F) ratio of 31/29, whereas patients with schizophrenia consisted of 63 subjects with an M/F ratio of 30/33. The mean age for healthy controls was 30 years, whereas that for patients with schizophrenia was 35 years. A standardized enzyme-linked immunosorbent assay (ELISA) technique was used to measure the concentration of IL-12p40 in all collected sera (*n* = 123). The mean IL-12p40 levels in patients with schizophrenia were significantly higher than in healthy controls (*p* = 0.002). Healthy females had a significantly higher concentration of IL-12p40 than healthy males (*p* = 0.009). Female patients with schizophrenia had significantly higher concentrations of IL-12p40 than their male counterparts (*p* < 0.001), healthy females (*p* = 0.018), and healthy males (*p* < 0.001), respectively. Male patients with schizophrenia had significantly higher concentrations of IL-12p40 than healthy males (*p* = 0.023). The study’s results suggest that IL-12p40 has a putative role as a potential marker in schizophrenia and that its elevation may participate in its pathogenesis. IL-12p40 may be included in a panel to be evaluated in the sera of patients with schizophrenia and an appreciation of its independent function is important for improving our understanding of both protective and pathogenic immune responses. Future research should aim to assess this interleukin and understand its role in other mental illnesses that share a similar etiology to schizophrenia.

## Introduction

Schizophrenia is a chronic and often debilitating mental disorder that affects approximately 1% of the world population. The onset of full-blown schizophrenia typically occurs in late adolescence or in early adulthood with a varied symptomatology, which comprises delusions, hallucinations, negativism, and cognitive scarcities (American Psychiatric Association, 2013). Even though several etiological dynamics still need to be clarified, interactions between genetic vulnerability and environmental stressors in the primary phases of life are vital in the advancement of schizophrenia (McDonald and Murray, 2000; Harrison and Weinberger, 2005). Along with genetic and neurodegenerative factors, inflammation has also been regarded as a major causative and/or contributing/mediating factor for schizophrenia occurrence (Fan et al., 2007).

Inflammation is the first reaction of the immune response, and it represents a complex reaction by the host to tissue injury such as infection or physical insult (Nathan, 2002). The main function of inflammation is to reestablish host homeostasis by allowing recovery from damage. Given that the adverse repercussions of the inflammatory progressions may be damaging to the host, they must be fast, precise, and self-limited (Na et al., 2014). Immune responses in the body are branded as either innate or adaptive. Innate immunity is chiefly formed of circulating effector cells (mast cells, phagocytes, natural killer cells, and microglia) and its key role is to rapidly eradicate pathogens in a non-specific way and to commence an adaptive immune response via exciting antigen-specific T and B lymphocytes (Na et al., 2014). Meanwhile, adaptive immunity explicitly distinguishes and recalls pathogens. T helper cells play a vital part in arbitrating the adaptive immune response. Some have proposed that naïve T helper cells develop into type 1 T helper cells (Th1) or type 2 T helper cells (Th2) in response to specific types of cytokines (Mosmann et al., 1986; Seder and Paul, 1994; D’Elios and Del Prete, 1998; Na et al., 2014). A cytokine, or interleukin (IL), is formed of tiny glycoproteins that intercede signal communications amongst several immune and neuronal cells throughout the immune response (Na et al., 2014).

Cytokines are formed via outlying immunocompetent cells, glial cells, and neurons (Woodroofe, 1995; Na et al., 2014). It is known that Th1 cells are involved in cellular immunity against intracellular bacteria and viruses, as well as in other autoimmune diseases such as multiple sclerosis and rheumatoid arthritis (Na et al., 2014). Contrastingly, Th2 cells direct humoral immunity against extracellular parasites and allergic reactions (Na et al., 2014).

Numerous studies have investigated alterations in peripheral cytokine secretion in schizophrenia (Kim and Maes, 2003; Kim, 2005; Stober et al., 2009). Although, the central nervous system (CNS) is somehow secluded from the peripheral immune system by the blood brain barrier (BBB), it is possible for a systemic peripheral cytokine to invade the BBB and reach the CNS under normal physiological conditions (Banks, 2005), via several mechanisms that comprise saturable transport (Banks et al., 1989; Osburg et al., 2002), disruption of the BBB (Quagliarello et al., 1991), and through the circumventricular organs that lack the BBB (Buller, 2001). Pro-inflammatory cytokines such as tumor necrosis factor-α (TNF-α), IL-6, IL-1β, and interferon-γ (IFN-γ), formed by persistently actuated macrophages and T lymphocytes have also been conveyed as immunological altered components in schizophrenia (Smith and Maes, 1995; Na et al., 2014).

IL-12p40 is identified as a constituent of the bioactive cytokines IL-12 and IL-23; however, it is not broadly acknowledged as having inherent functional activity (Trinchieri, 2003; Trinchieri et al., 2003; Hunter, 2005). Nevertheless, contemporary research has changed this view and backed an autonomous role for IL-12p40. It is incited excessively over the other subunits of IL-12 and IL-23 and may be present in a monomeric or homodimeric form (Mattner et al., 1993; Gillessen et al., 1995; Gately et al., 1996). It is most commonly valued for providing a negative feedback loop by competitively binding to the IL-12 receptor (Mattner et al., 1997). Nevertheless, IL-12p40 is a chemoattractant for macrophages and stimulates the migration of bacterially enthused dendritic cells (Shimozato et al., 2006). It is associated with several pathogenic inflammatory responses such as silicosis, graft rejection and asthma, but it has also been shown to be protective in a mycobacterial model (Abdi, 2002; Ozbey et al., 2008). Appreciating the sovereign function of IL-12p40 is vital to expand our understanding of both protective and pathogenic immune responses (Cooper and Khader, 2007). In patients with schizophrenia, plasma levels of IL-12 were investigated in two studies (Kim et al., 2002; Ozbey et al., 2008); these studies showed contradicting results regarding the IL-12 regulation. However, variations of the IL-12p40 have not been investigated for patients with schizophrenia yet.

In this article, we aimed to compute and assess IL-12p40 levels in the sera of individuals with schizophrenia from different provinces in Lebanon and compare it to controls.

## Materials and Methods

### Study Population

Ethical approval for this study was obtained from the Institutional Review Board of the Lebanese University, Beirut, Lebanon. Patients originated from different regions of Lebanon and were recruited from Nabih Berri Governmental University Hospital and Al-Fanar Psychiatric Hospital. Patients who met the Diagnostic and Statistical Manual of Mental Disorders (5th Edn; DSM–5; American Psychiatric Association, 2013) diagnostic criteria for schizophrenia were recruited. All interviews to assess diagnosis were carried out by a single trained clinical psychiatrist. These patients were treated with clozapine antipsychotic medication. A total of 113 specimens were received, although only 63 specimens, with a male/female (M/F) ratio of 30/33, were included in the study following proper enzyme-linked immunosorbent assay (ELISA) reproducibility (*n* = 63). Serum was collected after obtaining personal consent and stored in a freezer at -80°C.

Healthy controls were recruited from the same area as patients with schizophrenia and matched on demographics such as age, sex, and socioeconomic status. Out of 120 specimens provided, only 60 specimens with a M/F ratio of 31/29 were chosen on the basis of reproducibility in the ELISA test (*n* = 60). Control subjects were recruited through advertisement within the surrounding mental health clinics and hospitals. Informed, written consent was gained from all participants. Exclusion criteria for both groups included other psychiatric and neurological diagnoses. While psychometric testing was not performed for individual participants, all participating subjects were educated to at least a secondary school level in Lebanon or equivalent. None of the participants presented with systemic illness or signs of fever at the time of sample collection. Recruited healthy controls did not have a significant physical disability or disease. The demographic data (male: female ratio, age range, treatment duration, and daily dose) were also collected for each group. Neither patients with schizophrenia nor control subjects were current smokers or suffered from substance abuse/dependence. All recruited subjects were free of immunosuppressive medication. Notably, all patients who attended both hospitals were invited to participate in this study, and those who opted to participate did so on a voluntary basis; as such, we were unable to influence demographics, such as age and sex, of those who took part in this study.

### Treatment

The range of treatment duration for patients treated with clozapine was 6–19 years (with a mean treatment duration ± standard deviation of 9 ± 3.2 years). The range for the daily dose of clozapine medication was 100–400 mg per day (with a mean daily dose ± standard deviation of 184 ± 73 mg per day), *n* = 63. We chose to recruit patients treated with clozapine in the present study primarily for two reasons. Firstly, patients with a diagnosis of schizophrenia can often be non-compliant with their prescribed antipsychotic medication (Nosé et al., 2003). Those treated with clozapine are required to take the medication daily and monitor the plasma levels of clozapine regularly. Secondly, clozapine is considered a gold-standard therapy for treatment-resistant schizophrenia (Williams et al., 2002).

### Cytokine Analysis

The ELISA test was performed according to the user manual (RayBio Human IL-12p40; RayBiotech, Inc.). All reagents, samples and standards were brought to room temperature (18–25°C) before their use. Each microplate consisted of 96 wells (12 strips × 8 wells) coated with anti-human IL-12p40. Then, 100 μl of each standard recombinant human IL-12p40 sample were added into appropriate wells, covered, and incubated for 2.5 h at room temperature, as well as overnight at 4°C with gentle shaking. Solutions were discarded and the wells were washed four times each with 300 μl wash buffer. After the last wash, the entire remaining wash buffer was aspirated. The plates were inverted and blotted against clean paper towels. A 100 μl of 1x biotinylated anti-human IL-12p40 antibody were added to each well and incubated for 1 h at room temperature with gentle shaking. The solutions were discarded and washed four times. A 100 μl of 400-times diluted HRP-conjugated streptavidin were added to each well and incubated for 45 min at room temperature with gentle shaking. A 100 μl of Tetramethylbenzidine One-Step Substrate Reagent were added to each well and incubated for 30 min at room temperature in the dark with gentle shaking. Finally, 50 μl of Stop solution (0.2 M sulfuric acid) was added to each well and read immediately at an optical density of 450 nm. The minimum detectable dose of IL-12p40 was typically less than 10 pg/ml. This method provided high specificity as there is no cross-reactivity with the remaining cytokines. All specimens were tested twice and only those that were reproducible were included in the results. The samples of the cases and the healthy controls were processed at the same time and in parallel during the same ELISA.

### Statistical Analysis

All statistical analyses were performed with SPSS (Statistical Package for the Social Sciences) for Windows software version 23 (IBM SPSS, 2015). The Chi square test was used to compare qualitative variables. For quantitative variables, an independent samples *t*-test was used to determine whether the mean difference of IL-12p40 concentrations in the healthy control group and the group of patients with schizophrenia is statistically significantly different to zero. *p*-values < 0.05 were considered statistically significant for all analyses.

## Results

The average age of healthy controls was 30 years; ages ranged between 24 and 34 years. The average age of patients with schizophrenia was 35 years; ages ranged between 29 and 41 years. **Table 1** summarizes the demographic data of studied subjects.

**Table 1:** Demographics of patients with schizophrenia (*n* = 63) and healthy control subjects (*n* = 60).

Characteristics	Patients with Schizophrenia		Healthy Controls	
	Number	Percent	Number	Percent
**Sex**				
Male	30	47.6	31	51.7
Female	33	52.4	29	48.3
**Level of education**				
Secondary	50	79.4	48	80
Undergraduate	13	20.6	12	20
**Hospital**				
Al-Fanar Psychiatric Hospital	40	63.5	40	66.7
Nabih Berri Governmental University Hospital	23	36.5	20	33.3
	**Mean**	***SD***	**Mean**	***SD***
**Age (year)**				
Males	34	2.6	30	1.9
Females	36	2.4	30	2.2
**Treatment**				
Duration (year)	9	3.2	-	-
Clozapine (mg/day)	184	73	-	-

*SD, standard deviation*.

**Table 2** summarizes the IL-12p40 concentrations in controls and individuals with schizophrenia. The mean IL-12p40 levels in patients with schizophrenia were significantly higher than in healthy controls (*p* = 0.002). Healthy females had a significantly higher concentration of IL-12p40 than healthy males (*p* = 0.009). Female patients with schizophrenia had significantly higher concentrations of IL-12p40 than their male counterparts (*p* < 0.001), healthy females (*p* = 0.018), and healthy males (*p* < 0.001), respectively. Male patients with schizophrenia had significantly higher concentrations of IL-12p40 than healthy males (*p* = 0.023). We found no significant associations between IL-12p40 concentrations and other demographical factors.

**Table 2:** Concentration of IL-12p40 (ng/ml) in all studied individuals (*n* = 123).

Variables	Patients with Schizophrenia (*n* = 63)	Healthy Controls (*n* = 60)
Males	0.84 ± 0.11 Δ	0.43 ± 0.12
Females	1.77 ± 0.22 †	1.02 ± 0.18
Total	1.33 ± 0.14 ^∗^	0.72 ± 0.12

*All values are reported as mean ± standard error of mean. Statistical analyses were performed by using the independent samples *t*-tests († significant compared to males in each group and to healthy females; ^∗^ significant compared to total healthy controls; Δ significant compared to healthy males). Significance indicates a *p*-value < 0.05 and a 95% Confidence Interval*.

**FIGURE 1:**
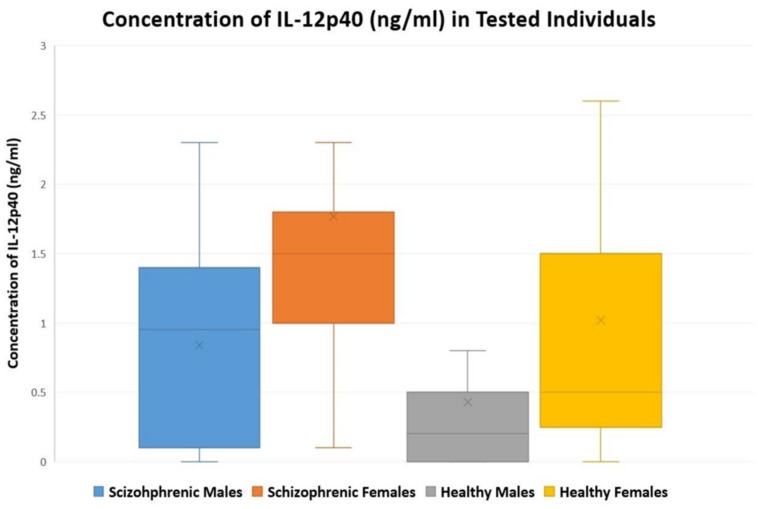
**A Box-and-Whisker plot showing the distribution of tested individuals versus concentration of IL-12p40 (ng/ml) in males and females of patients with schizophrenia (*n* = 63) and healthy controls (*n* = 60); outliers were excluded from the plot**.

**Table 3** summarizes the concentrations of IL-12p40 for all the individuals tested. In healthy controls, the concentration of IL-12p40 (in ng/ml) of males ranged between 0 and 2.7, while that of females ranged between 0 and 4.3. In patients with schizophrenia, the concentration of IL-12p40 in males ranged between 0 and 2.3; however, that of females ranged between 0.1 and 6.2. **Figure 1** depicts this distribution while excluding outliers.

**Table 3:** The distribution of tested individuals versus concentration of IL-12p40 (ng/ml) in male and female patients with schizophrenia and healthy controls (*n* = 123).

		Males	Females	Males	Females
		Patients with Schizophrenia (*n* = 63)		Healthy Controls (*n* = 60)	
Concentration of IL-12 p40 (ng/ml)	6.2		1		
	6.1				
	6				
	5.9				
	5.8				
	5.7				
	5.6				
	5.5				
	5.4				
	5.3				
	5.2				
	5.1				
	5		1		
	4.9				
	4.8				
	4.7				
	4.6				
	4.5				
	4.4				
	4.3				1
	4.2				
	4.1				
	4				
	3.9		1		1
	3.8				
	3.7				
	3.6				
	3.5				
	3.4		1		
	3.3		1		
	3.2				
	3.1				
	3				
	2.9				
	2.8				
	2.7			1	
	2.6				1
	2.5				
	2.4				
	2.3	1	1	1	
	2.2				2
	2.1	1	1		
	2			1	
	1.9	1			
	1.8		3		
	1.7		2		1
	1.6		2		
	1.5	2	4		2
	1.4	3	4		1
	1.3		1	1	1
	1.2	2	1		
	1.1	1			1
	1	4	3		
	0.9	2			1
	0.8		1	1	1
	0.7	1	1		
	0.6	1	2	1	1
	0.5			2	2
	0.4			1	2
	0.3		1	3	4
	0.2	3		6	1
	0.1	5	1	1	3
	0	3		12	3

## Discussion

This is the first study that measures pro-inflammatory cytokine levels in patients with schizophrenia in Lebanon, in addition to the Middle East and North Africa (MENA) region. Our results reveal a trend toward higher levels of IL-12p40 and, subsequently, IL-12 in patients with schizophrenia compared to controls.

The impact of sex on cytokine levels was examined. These data are in agreement with a previous study, suggesting that a raised level of cytokines in patients with schizophrenia predominates in females (O’Connell et al., 2013). Further, another current study observed that pro-inflammatory cytokines and body mass index (BMI) were higher in female patients compared to male patients and controls, aligning with the idea that visceral fat and altered adipocyte function could mechanistically explain elevated levels of pro-inflammatory cytokines in schizophrenia (O’Connell et al., 2014). Bjorling and Wang (2001) postulated that estrogen may modulate neurogenic inflammation by interacting with other substances and cells that participate in the pathogenesis of neurogenic inflammation, including substance P, bradykinin, and mast cells. Moreover, Hanamsagar and Bilbo (2015) stated that microglia play a crucial role in determining the onset and modulation of inflammation and thus sex differences in microglial function could explain, at least in part, differences observed in susceptibilities and outcomes of neurological disorders in men and women. In rodents, females have significantly more microglia with thick, long processes than males in sub-regions of the hippocampus as well as in the amygdala and parietal cortex (Schwarz et al., 2012). Mor et al. (1999) and Vegeto et al. (2001) added that, in terms of activation patterns, it has been shown that estrogen can regulate cytokine expression by microglia at basal level as well as in the presence of an inflammatory challenge. Testosterone, on the other hand, is known to have an inhibitory effect on glial activation (Barreto et al., 2007). Additionally, differences such as hormonal function, lifestyle and metabolic function may also play a role in explaining the raised levels of pro-inflammatory cytokines in females (O’Connell et al., 2014).

Several studies investigating chemokines that seemed to play an important role in modulating brain functions justify the bidirectional communications between nervous and immune system cells and their implications on psychiatric disorders (Ransohoff et al., 1996; Kronfol and Remick, 2000; Biber et al., 2002; Garver et al., 2003; Adler and Rogers, 2005; Cardona et al., 2008; Drexhage et al., 2008; Padmos et al., 2008). The relationship between several immune factors and the pathophysiology of schizophrenia has always been a subject of debate. Studies hypothesized that in patients with schizophrenia and major mood disorders (Garver et al., 2003; Drexhage et al., 2008; Padmos et al., 2008), a pro-inflammatory state of the cytokine network could induce psychopathologic symptoms and be involved in the pathogenesis and pathophysiology of these major mental illnesses. Increased serum concentrations of IL-2, IL-6, and IL-8 have been observed in patients with schizophrenia (Lin et al., 1998; Zhang et al., 2004). Moreover, a recent report has shown elevated IL-1β levels in the cerebrospinal fluids (CSFs) of the patients with drug-naïve schizophrenia (Söderlund et al., 2009). The elevation of pro-inflammatory cytokines in patients with schizophrenia helps us to understand why many patients have conjoint autoimmune diseases (Saetre et al., 2007; Dantzer et al., 2008).

Our results contradict those reported by Kim et al. (2002), which suggest that Th1 cytokines such as IL-2 and IL-12 are decreased in schizophrenia, but conform to those reported by Ozbey et al. (2008) that demonstrate a significant elevation of IL-12. Subsequent studies add that Th2 cytokines, such as IL-10, are augmented (Kim et al., 2002; Maes et al., 2002). Founded on the dichotomous notion of an adaptive immune response, the theory of Th1/Th2 imbalance was proposed as a potential mechanism (Schwarz et al., 2001). Another study by Miller et al. (2011) reported similar findings to ours. They established that pro-inflammatory cytokine levels are constantly augmented in patients with schizophrenia and stated that pro-inflammatory cytokines like IL-6, IL-12, TNF-α, IL-1β, and IFN-γ are elevated in the blood and CSF in initial-onset and acute-relapse patients with schizophrenia (Miller et al., 2011; Na et al., 2014). The outcomes of many recent publications have reinforced the function of pro-inflammatory cytokines in schizophrenia. Saetre et al. (2007) conveyed that an inflammation linked gene is augmented in schizophrenia. Additionally, Söderlund et al. (2009) reported that IL-1β is expressively augmented in the CSF of those with schizophrenia when compared to healthy volunteers.

Levels of pro-inflammatory cytokines have been shown to be increased not only in patients with schizophrenia but also in patients with bipolar disorder. After performing a comprehensive analysis of inflammatory molecules involved in schizophrenia and bipolar disorder, Dimitrov et al. (2011) speculated that dysregulation of a particular set of cytokines may lead to schizophrenia, while another set may lead to depression or bipolar disorder.

As described in **Figure 1**, the levels of IL-12p40 were moderately high in healthy individuals. Personal, familial, and societal stressors that affect Lebanese individuals could have contributed to these elevated cytokine levels (Fares et al., 2014; Hoteit and Fares, 2014). The socioeconomic and political situation in Lebanon, along with the regional instability and the numerous conflicts that the Lebanese people have witnessed, contributes to a higher degree of neuropsychological stress and anxiety (Fares and Fares, 2013; Fares et al., 2013a,b).

### Strengths and Limitations

This is the first study to measure pro-inflammatory cytokine levels in patients with schizophrenia in Lebanon and the MENA region. This study succeeds in showing that the levels of IL-12p40 are significantly elevated in patients with schizophrenia. However, the sample size and the lack of proper medical records (biochemical data and cardiovascular disease risk factors) for the participants can be considered as limitations of the study results. Cytokine levels in serum or plasma are confounded by a number of conditions, such as age, sex, socioeconomic status (O’Connor et al., 2009), metabolic syndrome and visceral obesity (Weisberg et al., 2003; Xu et al., 2003), smoking (De Leon et al., 2002), physical activity (Pledge et al., 2011), poor-rated self-health (Janszky et al., 2005; Christian et al., 2011), and medication (Miller et al., 2011). In our study, we succeeded in controlling many confounders as healthy controls were recruited from the same area as patients with schizophrenia; both groups were comparable in demographics such as age, sex, medical history, smoking history, and socioeconomic status.

### Future Research

For a greater understanding of the etiology and pathophysiology of schizophrenia, further studies in this field are urgently needed to widely assess the actual status of cytokines in a population with high predisposition to varied mental and psychiatric disorders. Additionally, therapeutic strategies for schizophrenia that target the inhibition of specific pro-inflammatory cytokine (such as IL-12p40) activation could be further explored. Future research should also aim to assess IL-12p40 and understand its role in other mental illnesses that share a similar etiology to schizophrenia.

## Conclusion

The current study examined the levels of IL-12p40 in 63 patients with schizophrenia treated with antipsychotic medication, as well as 60 healthy controls. Patients diagnosed with schizophrenia showed significantly elevated levels of IL-12p40 compared to controls. Healthy females had significantly higher concentrations than healthy males. Female patients with schizophrenia had a significantly higher concentration of IL-12p40 than their male counterparts and healthy controls, respectively. Male patients with schizophrenia had a significantly higher concentration of IL-12p40 than healthy males. The results of this study suggest that IL-12p40 has a putative role as a potential marker in schizophrenia and that its dysregulation may participate in the pathogenesis of schizophrenia. IL-12p40 may be included in a panel to be evaluated in the sera of patients with schizophrenia.

## Author Contributions

JF and YF designed the study, collected the data, did the statistical analyses and drafted the manuscript. All authors contributed to the analysis of the results. All authors revised the manuscript critically for important intellectual content and all authors again gave final approval of the version to be submitted. JF, FK, and YF are corresponding authors. YF is the primary investigator.

## Conflict of Interest Statement

The authors declare that the research was conducted in the absence of any commercial or financial relationships that could be construed as a potential conflict of interest.
